# Epithelial-myoepithelial carcinoma arising from the subglottis: a case report and review of the literature

**DOI:** 10.1186/s13256-016-0824-8

**Published:** 2016-02-27

**Authors:** Hun-Jae Oh, Nam-yong Do, Keun-Hong Kee, Jun-Hee Park

**Affiliations:** 1 Department of Otolaryngology-Head and Neck Surgery, Chosun University Medical School, 365 Pilmun-daero, Dong-gu, Gwanju, 501-717 South Korea; 2 Department of Pathology, Chosun University Medical School, Gwanju, South Korea

**Keywords:** Epithelial-myoepithelial carcinoma, Subglottis

## Abstract

**Background:**

Epithelial-myoepithelial carcinoma is an extremely rare disease that usually occurs in the parotid gland but can occur in a variety of sites such as the nasal cavity, paranasal sinus, and base of the tongue.

**Case presentation:**

We report a rare case of epithelial-myoepithelial carcinoma, which developed in the subglottic region. A 78-year-old Korean woman visited our hospital complaining of hoarseness, which had developed 1 month previously. Flexible laryngoscopy showed a round mass that blocked approximately 80 % of the tracheal diameter. Complete excision of the mass was carried out under general anesthesia, using a transoral approach. Epithelial-myoepithelial carcinoma was diagnosed following immunohistochemical analysis.

**Conclusions:**

We report a rare case of epithelial-myoepithelial carcinoma that occurred in the subglottic region. To the best of our knowledge, only one other case has been reported since this disease was first identified approximately 40 years ago.

## Background

Epithelial-myoepithelial carcinoma is an extremely rare disease, first reported by Donath *et al*. in 1972 and officially classified by the World Health Organization in 1991 [1–3]. As the term suggests, epithelial-myoepithelial carcinoma comprises two cell groups with distinct features, which are the key to diagnosis. It usually develops in major salivary glands such as the parotid gland, but infrequently occurs in the maxillary sinus, trachea, and larynx [2, 4, 5].

Epithelial-myoepithelial carcinomas in the subglottic region are very rare and to the best of our knowledge only one case has been reported to date [6]. We report a case of epithelial-myoepithelial carcinoma that developed in the subglottic region, together with a literature review.

## Case presentation

**Fig. 1:**
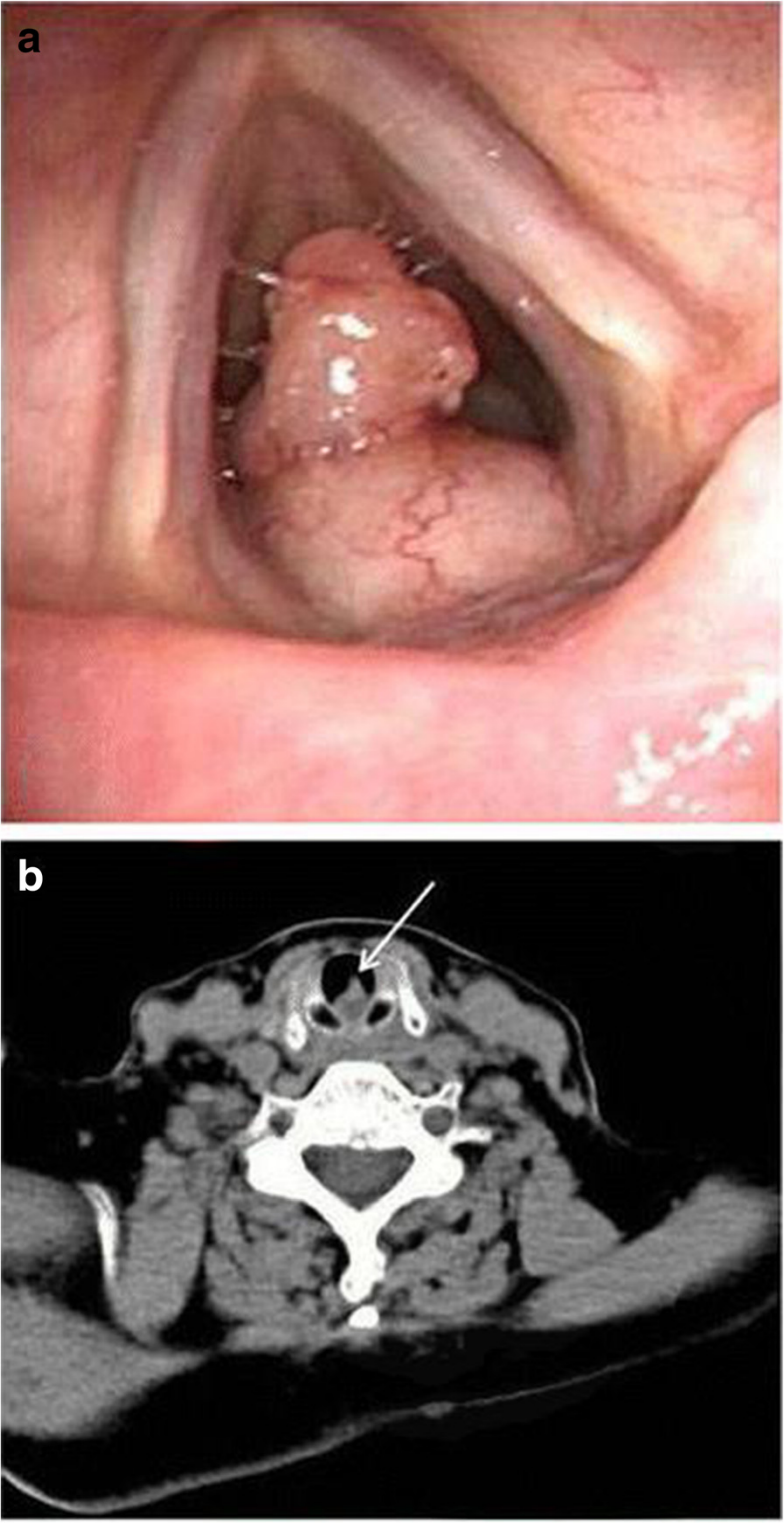
**a** A round protruding mass, 1 × 0.8 cm, below the interarytenoid space, and an approximately 0.8 × 0.8 cm protruding mass are visible. **b** A computerized tomography image demonstrated similar findings to those of the flexible laryngoscopy (*arrow*)

A 78-year-old Korean woman visited our hospital complaining of hoarseness, which had developed 1 month previously. Our patient had an unremarkable medical history except she was taking antihypertensive medication. Flexible laryngoscopy detected a mass approximately 1 cm in diameter on the posterior wall of her subglottic region that blocked approximately 80 % of her trachea (Fig. 1a). Neck computed tomography, without limiting her vocal cord movement, was performed owing to the suspected malignant tumor in her subglottic region. A well-localized 1 × 0.8 cm polypoid mass was detected in the posterior wall of her subglottic region (Fig. 1b).

Our patient underwent a bronchoscopic biopsy using a bronchus endoscope. Examination of the specimen showed the presence of epithelial and myoepithelial cells. Consequently, a tracheostomy and laryngeal microsurgery were performed under general anesthesia for a final biopsy and complete excision, with consideration given to the signs and symptoms of tracheal obstruction.

**Fig. 2:**
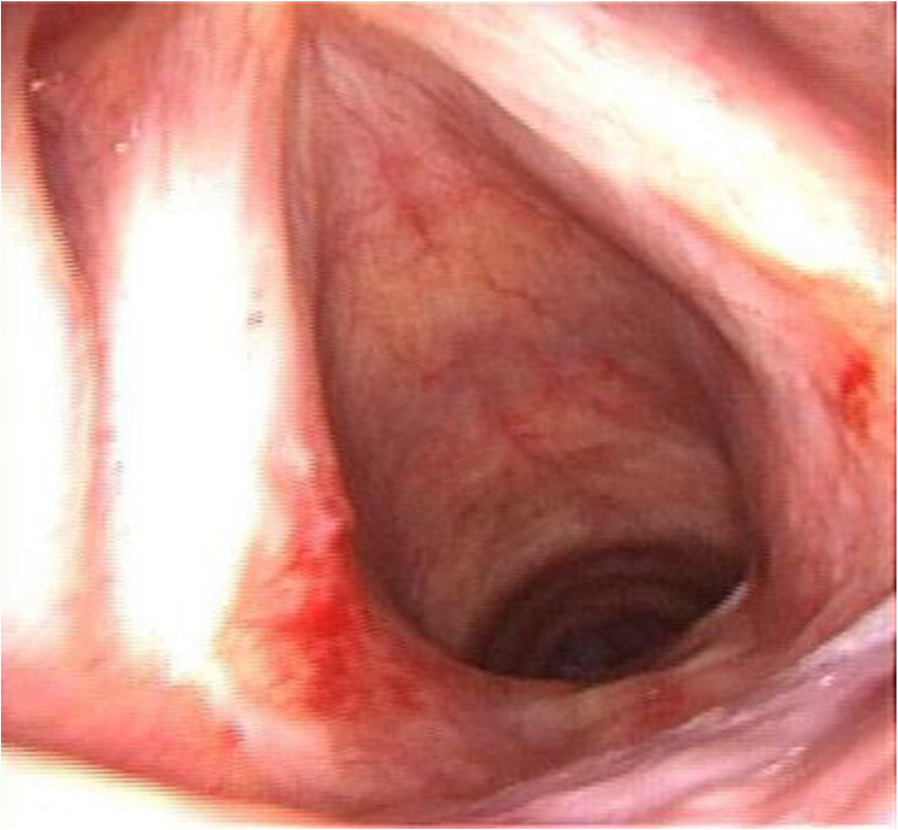
Complete excision of the masses using a carbon dioxide laser

A laryngeal microscopic examination revealed the round 1 × 0.8 cm protruding mass at the bottom of the interarytenoid space. Above this, the 0.8 × 0.8 cm mass protruded like a spire. Complete excision of the masses was possible using a carbon dioxide laser because there was no adhesion between the masses and the surrounding larynx (Fig. 2).

**Fig. 3:**
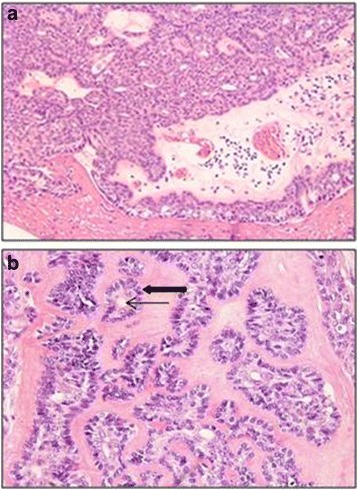
Histopathological appearance of the tumor. **a** Tumor cells are arranged in tubular, nested, and cord-like patterns (hematoxylin and eosin stain, ×100). **b** The duct-like structure is bi-layered with an inner layer comprising a single row of oval to round cells (thin arrow) and an outer layer of polygonal cells with clear cytoplasm (thick arrow; hematoxylin and eosin stain, ×200)

**Fig. 4:**
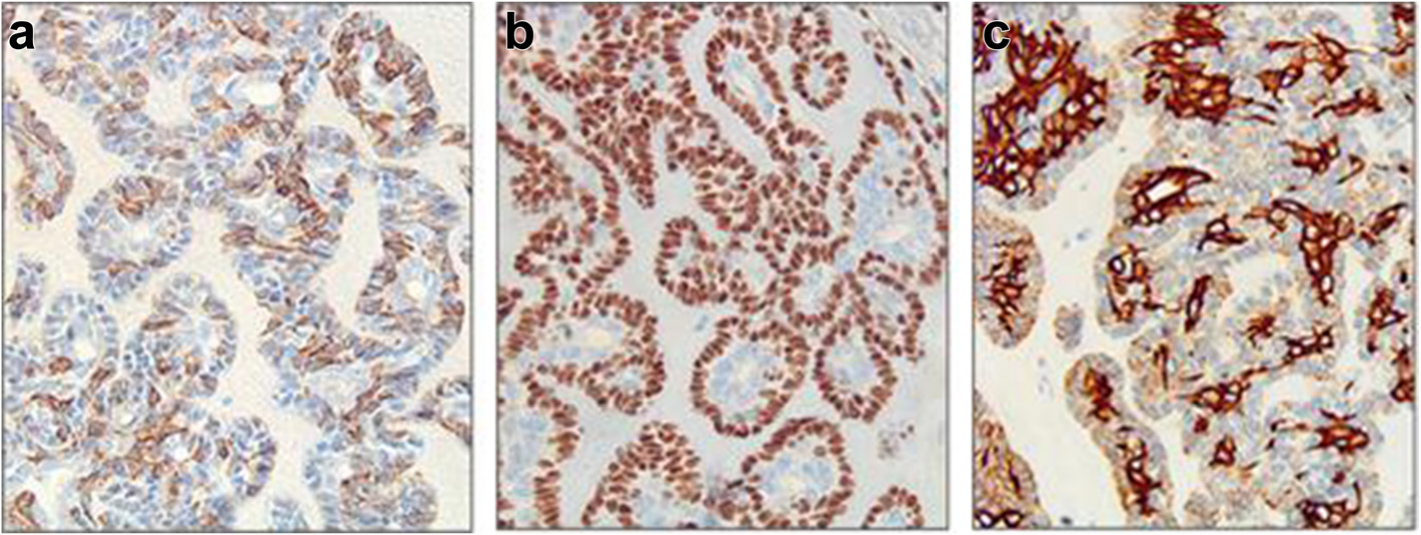
In immunohistochemical analysis, the neoplastic myoepithelial cells (cells of the outer layer) show reactivity for smooth muscle actin (**a**) and p63 (**b**), and the outer myoepithelial cell show weak positivity and inner epithelial cells show strong positivity for low molecular weight cytokeratin (**c**) (×400)

The gross findings of a histopathological examination were of ash-colored masses and a microscopic examination showed a tube-shaped structure composed of two layers. The inner layer was an oval or round monolayer of cells and the outer layer had polygonal cells with transparent cytoplasm (Fig. 3). Because the transparent myoepithelial cells in the outer layer showed weak-positive immunohistochemical staining for smooth muscle actin (SMA) and p63, and the epithelial cells were strongly positive for low molecular cytokeratin, epithelial-myoepithelial carcinoma was diagnosed (Fig. 4).

There were no complications, and our patient was discharged from hospital 10 days after surgery. Although additional radiation treatment was planned to prevent local recurrence, our patient refused this treatment. At the time of writing, her condition is being monitored by our outpatient clinic. There were no unusual findings 1 year after surgery.

## Discussion

A relatively rare disease, epithelial-myoepithelial carcinoma mostly develops in the parotid gland, but some reports claim that it also occurs in the nasal cavity, paranasal sinus, nasopharynx, bronchus, lung, lacrimal gland, submandibular gland, and base of the tongue [2, 4, 5]. To the best of our knowledge, only one other case of epithelial-myoepithelial carcinoma in the subglottic region has been reported previously [6].

It has been highlighted that epithelial-myoepithelial carcinoma develops more commonly in women and has a higher incidence in the fifth to eighth decades. The symptoms of epithelial-myoepithelial carcinoma vary depending on its anatomic position. When it intrudes into the parotid gland, the symptoms vary from subclinical tumentia to facial nerve palsy. When it encroaches into the larynx, as found in our case, various symptoms can occur depending on the carcinoma’s size, ranging from no symptoms to voice changes and respiratory obstruction [2, 4, 5].

Epithelial-myoepithelial carcinoma is diagnosed using an optical microscope and immunohistochemistry. On histology, epithelial-myoepithelial carcinoma is characterized by the presence of tubules with two distinct types of cells. The main histological features are epithelial cells in the inner layer of the lumen and myoepithelial cells surrounding the outer layer [2, 7]. These features are similar to those of intercalated ducts. Although it is helpful for diagnosis to confirm the presence of both cell groups with hematoxylin-and-eosin staining, the key to diagnosis involves clarifying the exact cell composition. In immunohistochemical analysis, myoepithelial cells in the outer layer are positive for calponin, p63 protein, glial fibrillary acidic protein, S-100 protein, and SMA. Epithelial cells in the inner layer are positive for cytokeratin-7 and epithelial membrane antigen [7]. In our case, the outer myoepithelial cells tested positive for SMA and p63, while the inner epithelial cells were positive for cytokeratin; hence, epithelial-myoepithelial carcinoma was diagnosed.

Other diseases that should be considered in the differential diagnosis of epithelial-myoepithelial carcinoma are pleomorphic adenoma, myoepithelial carcinoma, and adenoid cystic carcinoma [8]. Epithelial-myoepithelial carcinoma with a large amount of stroma resembles a mixed tumor, but invasiveness indicates the presence of a carcinoma [9]. The local morphological features of epithelial-myoepithelial carcinoma can be observed in adenoid cystic carcinoma and acinic cell carcinoma. Thus, these carcinomas should be widely excised to distinguish them from aggressive malignant tumors like epithelial-myoepithelial carcinoma.

Epithelial-myoepithelial carcinoma is regarded as a low-grade-malignancy tumor and the treatment protocol involves wide surgical excision with secure clear margins. Simple excision should be avoided because epithelial-myoepithelial carcinoma often has a destroyed epithelium [8, 10]. In the previous report, surgery was repeated several times because of local recurrence, but in our case, we completely removed the tumor by carbon dioxide laser surgery. Although postoperative radiotherapy is generally performed to reduce local recurrence, the effect of chemotherapy is not yet clear [2, 8]. It is known that tumors >4 cm are often associated with local recurrence. Therefore, when a large tumor is present, it is important to monitor the patient’s postoperative status in the outpatient clinic. Notably, a case was reported where distal metastasis was found near the facial nerves, kidneys, brain, and lungs, and another where distal metastasis was found 28 years after surgery [8, 11].

## Conclusions

The presented case relates to an epithelial-myoepithelial carcinoma originating from the subglottic region, where there has been only one similar case reported previously. Although there was no recurrence 1 year after surgical excision using the transoral approach, it is believed that continued examination, including laryngoscopy, is needed in the outpatient clinic. If recurrence is suspected, aggressive treatments such as surgery and radiation will be required.

## Consent

Written informed consent was obtained from the patient for publication of this case report and any accompanying images. A copy of the written consent is available for review by the Editor-in-Chief of this journal.
